# Retrospective analysis of the analgosedative efficacy and safety of midazolam combined with fentanyl in mechanically ventilated neonates

**DOI:** 10.3389/fped.2025.1647247

**Published:** 2025-09-10

**Authors:** Fangfang Lin, Shuidi Lin, Wenhong Cai, Yanli Ren

**Affiliations:** Department of Neonatology, Fujian Maternity and Child Health Hospital, Fuzhou, China

**Keywords:** neonate, mechanical ventilation, sedation, analgesia, midazolam, fentanyl

## Abstract

**Purpose:**

To compare the analgosedative efficacy and safety of the combination of midazolam and fentanyl with those of midazolam monotherapy in mechanically ventilated neonates.

**Materials and methods:**

We conducted a single-center retrospective analysis of mechanically ventilated patients in our neonatal intensive care unit from April 1, 2021 to December 1, 2024. After dividing patients into midazolam + fentanyl (M + F) and midazolam (M) groups according to their respective sedation/analgesia regimens, we conducted intergroup comparisons of pain scores, ventilator parameters, and vital signs 5 min pre-treatment and at post-treatment time points (1 h, 3 h, 12 h, 24 h, 48 h). We also compared pre-and post-treatment (3 h, 24 h, 48 h) non-invasive hemodynamic parameters; adverse reactions; and discharge outcomes of the two groups.

**Results:**

A total of 210 neonates were included, with 106 in the M + F group and 104 in the M group. N-PASS pain scores obtained 5 min pre-treatment were similar between the two groups (*P* > 0.05); however, N-PASS scores were significantly lower in the M + F than in the M group at each post-treatment time point (1 h, 3 h, 12 h, 24 h, 48 h) (all *P* < 0.05). Ventilator parameters (MAP, FIO_2_) obtained 5 min pre-treatment were similar (*P* > 0.05), but were significantly lower at each post-treatment time point (1 h, 3 h, 12 h, 24 h, 48 h) in the M + F group (all *P* < 0.05). Pre- and post-treatment (1 h, 3 h, 12 h, 24 h, 48 h) vital signs did not differ between the two groups (*P* > 0.05). Pre-and post-treatment (3 h, 24 h, 48 h) non-invasive hemodynamic parameters were similar between the two groups (*P* > 0.05). There were no significant intergroup differences in adverse reactions or discharge outcomes (*P* > 0. 05).

**Conclusion:**

The combination of midazolam and fentanyl relieved pain and reduced ventilator parameters more effectively than midazolam monotherapy, without increasing adverse reactions or worsening discharge outcomes among mechanically ventilated neonates.

## 1 Introduction

Mechanical ventilation is the most common and effective treatment of respiratory failure in neonatal intensive care units (NICUs). Notwithstanding, it can bring discomfort, pain, hemodynamic changes, increased oxygen consumption, metabolic disorders, and increased stress hormone levels that increase the risks of complications and mortality (1, 2). Therefore, the provision of analgesia to mechanically ventilated neonates is particularly important.

A guide to neonatal pain assessment and management was developed at the University of Arizona in 2016 (3) and the 2023 edition of the Evidence-based Guidelines for the Management of Neonatal Pain in China (4) proposed that the persistent pain caused by mechanical ventilation should be treated with sedation and analgesia. However, neither guideline recommended specific sedative and analgesic regimens (3, 4). Consequently, NICU clinicians have implemented various empiric analgosediative regimens for mechanically ventilated neonates (5–7).

Among sedative drugs, midazolam exhibits effective sedation, a short half-life, a rapid onset of action, no obvious accumulation after long-term use, and rapid penetration of the blood-brain barrier, facilitating its suitability for neonatal sedation (8, 9). Consequently, midazolam is widely used for sedation in critically ill neonates. However, although midazolam is a potent sedative, it does not provide analgesia (10). Because few clinical studies of analgesia among mechanically ventilated neonates have been completed, clinicians inevitably refer to regimens used in adults and children. The 2018 Guidelines for Sedation and Analgesia Treatment in Chinese Adult ICU clearly state that fentanyl is the preferred analgesic in the critical care of adults (11). Opioids are the primary drugs for managing the pain of critically ill patients in the ICU, among which morphine and fentanyl are used most often (12, 13). The most used analgesics in pediatric ICUs are morphine, acetaminophen, and fentanyl; the most frequently used analgosedative regimens for mechanically ventilated children combine midazolam and fentanyl (14, 15). Consequently, the combination of midazolam and fentanyl is the most often used regimen for the sedation and analgesia of mechanically ventilated neonates (16, 17).

The National Medical Products Administration of China approved the use of fentanyl in 1968. Its primary indication was the treatment of moderate to severe chronic pain in adults. However, fentanyl therapy of neonates remains off-label and is not well defined. Evidence regarding its safety, efficacy, adverse reactions, and long-term effects in neonates is limited. Therefore, the purpose of this study was to evaluate the efficacy and safety of midazolam combined with fentanyl in mechanically ventilated neonates.

## 2 Methods

### 2.1 Subjects

We conducted a retrospective analysis of analgosedative regimens given to neonates (age from 0 to 28 days, gestational age from 25 to 42 weeks) receiving mechanical ventilation in the NICU of our hospital from April 1, 2021 to December 1, 2024. This study was approved by the ethics committee of our hospital (Fujian Maternity and Child Health Hospital approval number 2025KY042). Inclusion criteria were (1) receipt of invasive mechanical ventilation; and (2) treatment with either midazolam combined with fentanyl (M + F group) or midazolam alone (M group). Exclusion criteria included (1) congenital malformation; (2) inherited metabolic diseases; (3) less than 24 h of mechanical ventilation; (4) surgical operations; and (5) receipt of other sedative or analgesic drugs. Patients were categorized retrospectively into either the M + F or M group according to their respective sedative and analgesic regimens.

### 2.2 Study methods

#### 2.2.1 Demographic and baseline clinical information

Gender, gestational age, birth weight, and pre-treatment age, underlying diseases, and modes of respiratory support were obtained from medical record review. All patients were hospitalized in the same NICU. Pain scoring was conducted by trained nurses with NICU qualifications. Subjective evaluations did not differ between clinical care teams.

#### 2.2.2 Sedative/analgesic drug administration

All neonates undergoing mechanical ventilation were scored with the N-PASS scale during medication. Our department is comprised of five clinical teams that utilize varied clinical practices of analgosedation based on the conceptual outlooks and clinical experience of the participating physicians. Mechanically ventilated newborns for whom analgosedation was indicated by pain scores received either midazolam monotherapy or a midazolam + fentanyl combination regimen at the discretion of the patient's clinical care team. In our retrospective analysis, patients who had received midazolam monotherapy were designated as the midazolam group (Group M), and those who had received midazolam combined with fentanyl comprised the midazolam + fentanyl (Group M + F). Because our study's retrospective design introduced risks of selection bias and confounding variables, we excluded patients who had undergone surgical operations or had developmental malformations. Pre-treatment pain scores, gestational age, ventilator support patterns, and primary diseases of the two groups were compared to the baseline values of the control group (no routine analgosedation for the indication of mechanical ventilation).

Midazolam doses in the monotherapy regimen were administered (Drug Batch Number refer to the appendix) by continuous intravenous pump infusion at a rate of 1 μg/kg/min and titrated according to its sedative and analgesic effect to a maximum dose of 6 μg/kg/min. Because fentanyl therapy of neonates constitutes an off-label use in China, the Pharmaceutical Affairs Committee of our hospital approved the use of fentanyl in newborns in 2021. We obtained informed consent from family members before fentanyl administration. Fentanyl was administered by continuous intravenous pump infusion at a rate of 0.5 μg/kg/h and titrated according to its analgesic effect. The maximum doses of midazolam and fentanyl used in the M + F regimen were 6 μg/kg/min and 5 μg/kg/h, respectively.

#### 2.2.3 Clinical assessment

The Neonatal Pain, Agitation and Sedation Assessment Scale (N-PASS) (18, 19) was used for the quantitative assessment of pain. N-PASS scores; physiologic and ventilator parameters (oxygen saturation [SpO_2_], heart rate [HR], mean arterial blood pressure [MABP], fraction of inspired oxygen [FIO_2_], mean airway pressure [MAP]) were recorded 5 min pre-treatment and at 1 h, 3 h, 12 h, 24 h, and 48 h post-treatment time points. Noninvasive hemodynamic parameters [cardiac index [CI], cardiac output [CO], systemic vascular resistance index [SVRI]] were recorded 5 min before treatment and at 3 h, 24 h, and 48 h post-treatment time points. Noninvasive hemodynamic parameters were measured with an ultrasonic cardiac output monitor. Medical records were reviewed to determine duration of mechanical ventilation, cumulative doses of midazolam and fentanyl, and the positive muscle strength score before medication assessment. The positive muscle strength score was calculated as follows: dopamine (ug/kg.min) + dobutamine (ug/kg.min) + 100× millinol (ug/kg.min) + 100× epinephrine (ug/kg.min) + 100× norepinephrine (ug/kg.min) + 10,000 × vasopressin (U/kg). min). Additionally, adverse events, complications, and discharge outcomes were recorded. The attending physician determined the timing, dosage, and adjustment of vasoactive positive inotropic drugs based on the child's condition, blood pressure, HR, capillary filling time, and echocardiographic results.

#### 2.2.4 Definitions of adverse events, complications, and outcomes

Urinary retention was diagnosed if B-ultrasound examination revealed a full bladder that the neonate could not empty normally. Hypotension was defined as an MABP of <40 mmHg in full-term infants and <30 mmHg in premature infants, accompanied by manifestations of insufficient tissue perfusion. Bradycardia was defined as a HR <100 bpm. Respiratory depression was defined as a respiratory rate <30 breaths per min. Withdrawal reaction was defined by convulsions, muscle tremors, hyperreflexia, or continuous crying after drug withdrawal and unrelated to infection or craniocerebral disorders. Hepatic dysfunction was defined by elevations of serum ALT >100 U/L and/or AST >40 U/L; renal dysfunction was defined by significant elevations of serum creatinine (>1.5 mg/dl) and urea nitrogen (>11 mmol/L) and urine output <1 ml/kg/h. We monitored the incidence of necrotizing enterocolitis (stage II or above), periventricular leukomalacia, and intraventricular hemorrhage (III–VI). Discharge outcomes were categorized as either Recovery (resolution of primary disease, stable vital signs, and SpO_2_ > 90% without oxygen supplementation); Improvement [improvement of primary disease, but with a requirement for continuous or intermittent oxygen supplementation via nasal catheter (FIO_2_ < 30%)]; or Unresolved (primary disease has not improved substantially, continued ventilator requirement, and high risk of mortality).

#### 2.2.5 Statistical methods

We employed the SPSS23.0 statistical software package. After testing all data for normality, those conforming to normal distribution were expressed as mean ± standard deviation. The independent sample *t*-test was used for comparisons between two groups, and repeated measures analysis of variance was used for intra-group comparison. Data featuring non-normal distribution were expressed by median (quartile) [M (Q_1_, Q_3_)], with intergroup comparisons performed by the Mann–Whitney U rank sum test. The *χ*^2^ test was used for intergroup comparisons of count data expressed as frequencies and percentages. *P* < 0.05 was considered statistically significant.

## 3 Results

### 3.1 Demographic and baseline clinical characteristics

A total of 210 neonates were included, with 106 cases in the M + F group and 104 cases in the M group. The M group comprised 68 males and 38 females. with a mean gestational age of 34.84 ± 4.86 weeks; median age of 1.00 days, and mean body weight of 2.87 ± 1.16 kg. The M group consisted of 70 males and 34 females with a mean gestational age of 35.80 ± 3.56 weeks, a median age of 1.00 days, and a mean body weight of 2.52 ± 0.99 kg. There were no significant intergroup differences in gender, weight, gestational age, age in days, or respiratory support (all *P* > 0.05). *P* values of gender, weight, gestational age, age in days, and respiratory support were 0.603, 0.086, 0.099, 0.945, and 0.793, respectively. Additionally, baseline clinical characteristics, including underlying diseases (such as pneumonia, pulmonary hyaline disease, pulmonary hemorrhage) were similar between the two groups (all *P* > 0.05) (Table 1). All patients underwent a Neonatal Critical Illness Score assessment. Children with a score of 70 (extremely critical condition) were excluded to avoid confounding due to differences in initial disease severity.

**Table 1:** Demographic and baseline clinical data.

Indicator	Group		Statistics	*P* Value
	M + F (*n* = 106)	M (*n* = 104)		
Gender (%)	n (%)	n (%)	x^2^ = 0.232	0.630
Male (*n* = 138)	68 (49.3)	70 (50.7)		
Female (*n* = 72)	38 (52.8)	34 (47.2)		
Baseline Information	Mean ± SD	Mean ± SD		
Gestational age (weeks)	34.84 ± 4.86	35.80 ± 3.56	t = 1.659	0.099
Birth weight (kg)	2.47 ± 1.17	2.71 ± 0.80	t = 1.728	0.086
	M [Q_1_, Q_3_]	M [Q_1_, Q_3_]		
Age (days)	1.00 (1.00, 1.00)	1.00 (1.00, 1.00)	Z = 0.069	0.945
Modes of respiratory support	n (%)	n (%)	*χ*^2^ = 0.069	0.793
SIMV	60 (51.3)	57 (48.7)		
HFOV	46 (49.5)	47 (50.5)		
Underlying diseases *n* (%)	n (%)	n (%)		
Pneumonia	41 (48.2)	44 (51.7)	χ^2^ = 0.287	0.592
Pulmonary hemorrhage	9 (45.0)	11 (55.0)	χ^2^ = 0.265	0.607
Pulmonary hyaline disease	34 (55.7)	27 (44.3)	χ^2^ = 0.952	0.329
Meconium aspiration syndrome	7 (43.8)	9 (56.2)	χ^2^ = 0.313	0.576
Persistent pulmonary hypertension	3 (50.0)	3 (50.0)	χ^2^ = 0.001	0.981
Septic shock	5 (55.5)	4 (45.5)	χ2 = 0.097	0.755
Pneumothorax	3 (60)	2 (40)	χ^2^ = 0.186	0.666
BPD	1 (33.3)	2 (66.6))	χ^2^ = 0.358	0.550
NEC	3 (60)	2 (40)	χ^2^ = 0.186	0.666

M + F, midazolam + fentanyl group; M, midazolam group; SIMV, synchronized intermittent mandatory ventilation; HFOV, high-frequency oscillatory ventilation; BPD, bronchopulmonary dysplasia; NEC, necrotizing enterocolitis.

### 3.2 Pain score

**Figure 1:**
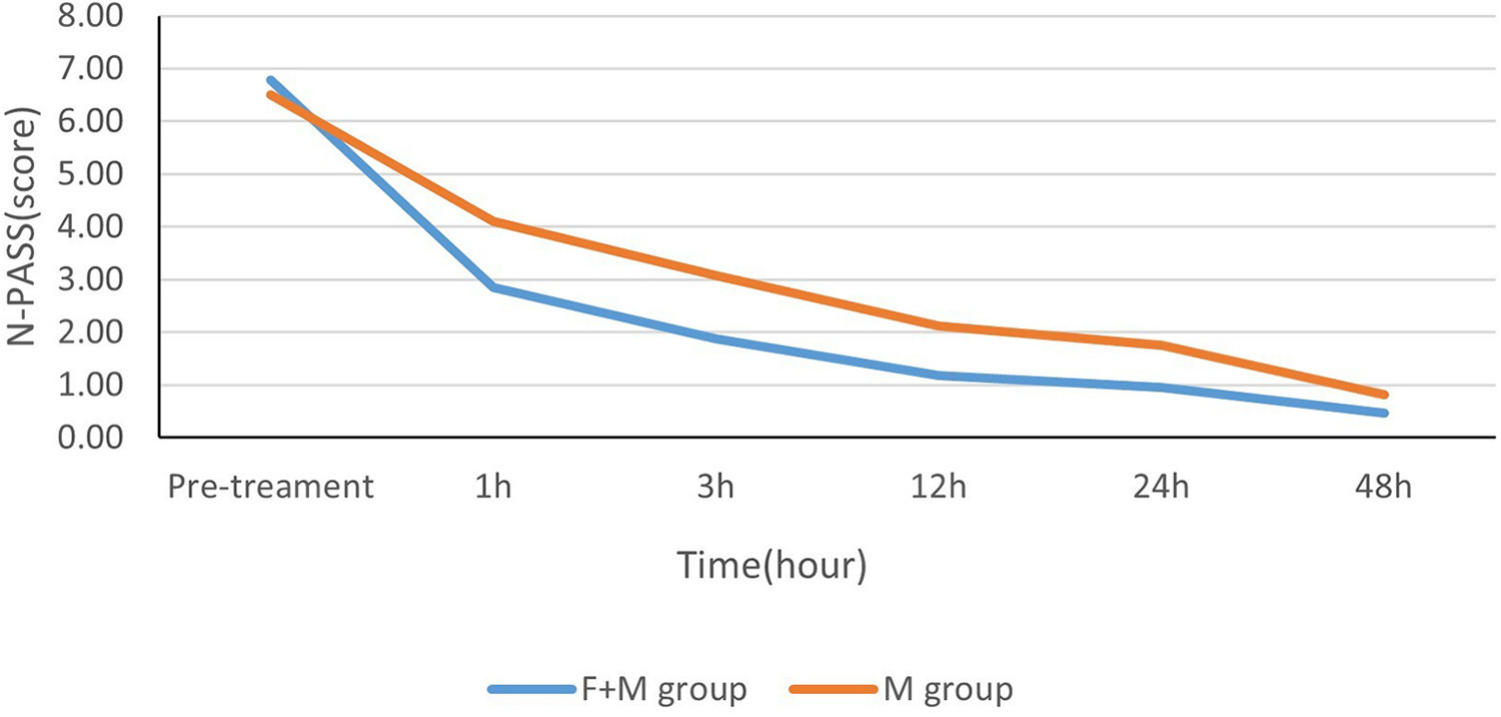
Intergroup comparison of N-PASS scores at pre- and post-treatment timepoints.

Median pretreatment N-PASS scores in the M + F and M groups were similar (7 and 6, respectively) (*P* = 0.129 > 0.05). The median N-PASS scores at each post-treatment each time point (1 h, 3 h, 12 h, 24 h, 48 h) were lower in the M + F group than in the M group (2.40,1.91,1.9, 1.0, and 0.0 in the M + F group and 4.0, 3.0, 2.0, 1.42, and 0.82 in M group, respectively; *P* values at each post-treatment time point were 0.000, 0.000, 0.000, 0.000, and 0.002, respectively) (Figure 1 and Table 2).

**Table 2:** Comparison of N-PASS scores at different time points.

Group	Pre-treatment	1 h post-treatment	3 h post-treatment	12 h post-treatment	24 h post-treatment	48 h post-treatment
	M [Q_1_, Q_3_]	M [Q_1_, Q_3_]	M [Q_1_, Q_3_]	M [Q_1_, Q_3_]	M [Q_1_, Q_3_]	M [Q_1_, Q_3_]
M + F	7.00 (6.00, 8.00)	2.40 (2.00, 4.00)	1.91 (1.00, 2.34)	1.00 (1.00, 1.65)	1.00 (0.21, 1.15)	0.00 (0.00, 1.00)
M	6.00 (6.00, 7.00)	4.00 (3.00, 5.00)	3.00 (2.00, 4.00)	2.00 (1.00, 3.00)	1.42 (1.00, 2.47)	0.82 (0.00, 1.00)
Z Value	1.519	6.53	5.687	5.563	5.001	3.062
*P* Value	0.129	0.000	0.000	0.000	0.000	0.002

### 3.3 Physiological and ventilator parameters

#### 3.3.1 Physiological parameters

Pre-treatment physiological parameters were similar between the two groups (all *P* > 0.05) (Table 3). Means and standard deviations of pre-treatment values of the M + F and M groups were SpO_2_ 92.16 ± 2.59% and 91.56 ± 3.82%; HR 147.01 ± 16.86 bpm and 144.94 ± 13.45 bpm; and MABP 43.30 ± 7.63 mmHg and 44.81 ± 6.07 mmHg, respectively. Pretreatment *P* values for interaction of SpO_2_, HR, MABP of the two groups were 0.182, 0.282 and 0.112, respectively.

**Table 3:** Repeated measures ANOVA of physiological and ventilator parameters.

Group	Date	Sp02 (%)	HR (bpm)	MABP (mm Hg)	FIO_2_ (%)	MAP (cm H_2_O)
		Mean ± SD	Mean ± SD	Mean ± SD	Mean ± SD	Mean ± SD
M + F	Pre-treatment	92.16 ± 2.59	147.01 ± 16.86	43.30 ± 7.63	53.82 ± 21.39	11.54 ± 2.94
	1 h post-treatment	93.69 ± 1.25	146.68 ± 14.63	42.90 ± 6.16	44.25 ± 18.47	10.95 ± 2.32
	3 h	93.54 ± 4.17	143.98 ± 15.55	43.89 ± 6.05	42.24 ± 17.63	10.67 ± 2.30
	12 h	93.64 ± 1.77	145.85 ± 16.21	43.21 ± 5.52	38.71 ± 16.13	8.20 ± 1.98
	24 h	93.80 ± 1.47	143.75 ± 16.86	44.67 ± 7.72	35.74 ± 15.96	8.06 ± 1.95
	48 h	94.69 ± 1.25	144.30 ± 16.95	45.91 ± 7.30	33.54 ± 15.01	7.91 ± 1.87
M	Pre-treatment	91.56 ± 3.82	144.94 ± 13.45	44.81 ± 6.07	49.29 ± 18.39	10.74 ± 3.07
	1 h post-treatment	93.38 ± 1.93	142.76 ± 12.71	44.36 ± 5.24	49.29 ± 18.38	10.58 ± 2.87
	3 h	93.55 ± 1.80	143.54 ± 13.44	44.55 ± 5.36	48.57 ± 19.04	10.33 ± 2.91
	12 h	93.56 ± 2.07	143.57 ± 13.16	44.68 ± 4.91	47.16 ± 19.23	10.13 ± 2.87
	24 h	93.95 ± 1.96	142.34 ± 13.58	45.23 ± 7.72	43.96 ± 19.56	9.87 ± 2.81
	48 h	93.95 ± 1.95	140.39 ± 14.00	46.98 ± 5.48	42.58 ± 19.18	9.65 ± 2.65
Overall coefficient of analysis		0.593	0.771	0.872	0.351	0.476
F and *P* values between groups		1.907, 0.169	2.168, 0.142	3.120, 0.079	5.374, 0.021	3.943, 0.048
F and *P* values of time		33.931, <0.001	3.380, <0.010	11.480 <0.001	86.207, <0.001	226.296, <0.001
F and *P* values for interaction		1.496, 0.215	0.935, 0.441	0.500, 0.752	23.319, <0.001	88.116, <0.001

SpO_2_, oxygen saturation; HR, heart rate; bpm, beats per minute: MABP, mean arterial blood pressure; FIO_2_, fraction of inspired oxygen; MAP, mean airway pressure.

There were no significant intergroup differences of physiological indexes and the interaction between the two groups at each post-treatment time point (all *P* > 0.05) (Table 3). The F values for interaction of SpO_2_, HR, MABP at each post-treatment time point of the two groups were 1.496, 0.935 and 0.500, with *P* values of 0.215, 0.441 and 0.752, respectively.

#### 3.3.2 Ventilator parameters

Pre-treatment ventilator parameters were similar between the two groups (all *P* > 0.05) (Table 3). Mean pretreatment values and standard deviations in the M + F and M groups were FIO_2_ 53.82 ± 21.39% and 49.29 ± 18.38%; and MAP 11.54 ± 2.94 cmH_2_O and 10.74 ± 3.07 cmH_2_O, respectively. *P* values for interaction of FIO_2_ and MAP at each post-treatment time point of the two groups were 0.101 and 0.053, respectively.

FIO_2_ and MAP values differed significantly between the two groups at each post-treatment time point (1 h, 3 h, 12 h, 24 h, 48 h) (all *P* < 0.05) (Table 3). The intergroup F values of FIO_2_ and MAP at each post-treatment time point were 5.374 and 3.943, respectively, with respective *P* values of 0.021 and 0.048. The intergroup F values for the interaction of FIO_2_ and MAP at each post-treatment time point were 23.319 and 88.116, with *P* values of <0.001 and <0.00, respectively.

#### 3.3.3 Non-invasive hemodynamic parameters

Pre-treatment non-invasive hemodynamic parameters of CI, CO, SVRI did not differ significantly between the two groups (all *P* > 0.05) (Table 4). Pre-treatment mean values and standard deviations in the M + F and M groups were CI 0.61 ± 0.23 L/min and 0.66 min ± 0.23 L/min; CO 3.65 ± 1.09 L/min/m^2^ and 3.28 ± 0.89 L/min/m^2^; and SVRI 1033.30 ± 275.82 dscm5m^2^ and 1096.38 ± 319.37 dscm5m^2^, respectively. *P* values for the interaction of CI, CO, and SVRI of the two groups were 0.136, 0.560, and 0.184, respectively.

**Table 4:** Repeated measures ANOVA of noninvasive hemodynamic parameters.

Group	Time	CO (L/min)	CI (L/min/m^2^)	SVRI (dscm^5^m^2^)
		Mean ± SD	Mean ± SD	Mean ± SD
M + F	Pre-treatment	0.61 ± 0.23	3.65 ± 1.09	1033.30 ± 275.82
	3 h post-treatment	0.59 ± 0.21	3.43 ± 0.74	1059.74 ± 263.18
	24 h	0.63 ± 0.28	3.71 ± 0.75	1051.28 ± 269.84
	48 h	0.63 ± 0.27	3.70 ± 0.94	1153.38 ± 233.96
M	Pre-treatment	0.66 ± 0.23	3.28 ± 0.89	1096.38 ± 319.37
	3 h post-treatment	0.65 ± 0.22	3.24 ± 0.62	1120.36 ± 224.67
	24 h	0.68 ± 0.21	3.47 ± 0.64	1141.02 ± 194.73
	48 h	0.69 ± 0.19	3.47 ± 0.50	1251.82 ± 256.92
Overall coefficient of analysis		0.965	0.824	0.913
F and *P* values between groups		3.673, 0.057	3.905, 0.051	4.832, 0.051
F and *P* values of time		5.871, 0.001	3.544, 0.022	6.378, 0.001
F and *P* values for interaction		0.420, 0.732	0.374, 0.732	0.155, 0.695

Post-treatment values of CI, CO, SVRI did not differ significantly between the two groups (all *P* > 0.05) (Table 4). The intergroup F values of CI, CO, and SVRI were 3.673, 3.905, and 4.832 respectively, with respective *P* values of 0.057, 0.051, and 0.051. The F values for the interaction of CI, CO, and SVRI of the two groups at each post-treatment time point were 0.420, 0.374, and 0.155, respectively, with respective *P* values of 0.732, 0.732, and 0.695.

#### 3.3.4 Duration of mechanical ventilation, adverse reactions and discharge outcomes

The median of duration of mechanical ventilation in the M + F and M groups were 5 and 4, respectively, and did not differ significantly (*P* *=* 0.228 > 0.05). Median cumulative midazolam doses were 2.88 (1.44, 6.79) mg/kg and 6.94 (2.88, 12.57) mg/kg in the M + F and M groups, respectively, and differed significantly between the two groups (*P* = 0.000 < 0.05). The vasoactive inotropic scores were 12.00 (8.00, 16.00) and 16.00 (8.00, 16.00) in the M + F and M groups respectively, and did not differ significantly between the two groups (*P* = 0.275 > 0.05). There were no significant intergroup differences in adverse reactions, complications, or discharge outcomes (*P* > 0.05) (Table 5).

**Table 5:** Duration of mechanical ventilation, adverse reactions, and discharge outcomes.

Indicator	Group		Statistics	*P* Value
	M + F (*n* = 106)	M (*n* = 104)		
	M [Q_1_, Q_3_]	M [Q_1_, Q_3_]		
Duration of mechanical ventilation (d)	5.00 (3.00, 8.25)	4.00 (3.00, 6.00)	Z = 1.206	0.228
Cumulative midazolam dose	2.88 (1.44,6.79)	6.94 (2.88,12.57)	Z = 4.334	0.000
Vasoactive inotropic score	12.00 (8.00,16.00)	16.00 (8.00,16.00)	Z = 1.091	0.275
Adverse reactions (%)	n (%)	n (%)		
Urinary retention	3 (50.0)	3 (50.0)	χ^2^ = 0.000	1.000
Hypotension	5 (45.5)	6 (54.5)	χ^2^ = 0.117	0.732
Bradycardia	4 (44.4)	5 (55.6)	χ^2^ = 0.001	0.977
Respiratory depression	1 (25)	3 (75)	χ^2^ = 0.275	0.600
Withdrawal reaction	2 (66.67)	1 (33.33)	χ^2^ = 0.000	1.000
Hepatic and renal dysfunction	13 (59.1)	9 (40.9)	χ^2^ = 0.731	0.393
Complications (%)				
NEC	5 (33.3)	8 (66.6)	χ^2^ = 0.800	0.371
Above Grade 3	6 (66.7)	4 (33.3)	χ^2^ = 0.381	0.537
PVL	5 (83.33)	1 (16.67)	χ^2^ = 1.486	0.223
Discharge outcomes			χ^2^ = 2.097	0.350
Recovered	96 (50.3)	97 (49.7)		
Improved	4 (44.4)	5 (55.6)		
Ineffective	6 (75.0)	2 (25.0)		

d, days; NEC, necrotizing enterocolitis; PVL, periventricular leukomalacia; IVH, intraventricular hemorrhage.

## 4 Discussion

Persistent neonatal pain can lead to cerebral cortical thinning and psychomotor retardation by causing hyperalgesia and hypoalgesia and consequent upregulation of pathogenic signaling pathways in the central nervous system (20, 21). The Expert Consensus on Analgesic and Sedative Treatment in Chinese Pediatric Intensive Care Unit recommends the combined use of sedatives and analgesics to maximize clinical benefits while reducing drug doses (22). The most used analgosedative drug regimens combine an opioid with a benzodiazepine delivered by continuous intravenous pump infusion (22). However, evidence regarding the safety, administration mode, dosage, and long-term effects of fentanyl in neonates is limited.

Yang Jingyue (23) et al. showed that the combination of midazolam and fentanyl in mechanically ventilated premature infants relieved pain and reduced the man-machine counteraction more effectively than midazolam alone. Combinations of midazolam with either fentanyl or remifentanil reduced ventilator parameters and shortened the duration of mechanical ventilation in neonates with respiratory distress syndrome (24, 25). Our study revealed that the pain scores of the M + F group were lower than those of the M group. Moreover, our findings of statistically significant differences in ventilator parameters (FiO_2_, MAP) suggest that the midazolam and fentanyl combination alleviates pain and decreases ventilator parameters more effectively than midazolam monotherapy in mechanically ventilated neonates, thereby reducing pulmonary injury, consistent with the results of other studies.

Although sedative and analgesic drugs can facilitate mechanical ventilation and relieve pain, they affect blood pressure, heart rate, and hemodynamics. Wang Lulu reported that the combination of midazolam and fentanyl outperformed the control regimen in the improvement of MABP, respiration, HR, and SpO_2_ in mechanically ventilated neonates and stabilized hemodynamic status, and did not increase adverse reactions such as urinary retention, hypotension, respiratory depression, withdrawal reaction, and hepatic and renal dysfunction (26). This study disclosed no statistically significant intergroup differences in hemodynamic parameters (CI, CO, SVRI). We suggest that the addition of fentanyl to midazolam, while improving ventilator settings (FIO_2_ and MAP), does not affect hemodynamic parameters (CI, CO, SVRI); does not increase hemodynamic instability, and does not increase the risk of complications such as intracranial hemorrhage and heart failure. Meanwhile, this study showed no significant intergroup differences in physiological indicators such as SpO_2_, MABP, and HR, indicating that the addition of fentanyl to midazolam did not affect these parameters. We propose that the discrepancies between our results and those of the Wang Lulu's study are because the control group in Wang Lulu's study did not receive routine analgosedation. This reflects the necessity of sedation and analgesia during mechanical ventilation.

A systematic review of neonatal fentanyl therapy comprised of studies published over the past 20 years showed that its use did not increase mortality or ventilation time, nor did it increase the incidence of bronchopulmonary dysplasia, necrotizing enterocolitis, intraventricular hemorrhage, periventricular leukomalacia, or sepsis. Fentanyl is considered safe for use in neonates (27). Another analysis involving 823 newborns from 13 independent studies showed that fentanyl lowered pain scores compared with placebo, but its effects on apnea, hypotension, and decreased heart rate were unclear (28).

This study found that the midazolam and fentanyl combination regimen did not increase adverse reactions such as urinary retention, hypotension, respiratory depression, withdrawal reactions, and liver and kidney dysfunction. This finding is similar to previously reported results.

We propose that the combination of midazolam and fentanyl not only reduces pain, pathophysiologic reactions to pain, ventilator parameters and consequent barotrauma, but may improve hemodynamic stability compared to midazolam monotherapy. The combination therapy did not destabilize vital signs such as SpO_2_, MABP and HR, nor did it increase complications ok adverse reactions. Therefore, we recommend midazolam + fentanyl combination therapy for the analgosedation of mechanically ventilated neonates.

A systematic review of 2023 newborns (with a minimum gestational age of 22 weeks) demonstrated that compared with placebo, no drug, or other analgesics or sedatives; fentanyl monotherapy had no significant effect on duration of hospital stay; mortality; or the incidence of necrotizing enterocolitis, intraventricular hemorrhage, and periventricular leukomalacia; as well as neuropsychological development monitored at 18–24 months and 5–6 years of age (29).

The long-term effects of neonatal fentanyl exposure on neurophysiological development are undetermined. Neurodevelopmental and social evaluations of preterm infants exposed to fentanyl found no significant independent correlation between the cumulative dose of fentanyl and intelligence quotient, language ability, and executive function after 5 years of follow-up (30–32). Notwithstanding, a multicenter cohort study of 936 extremely premature infants associated prolonged exposures (more than 7 days) to both opioids and benzodiazepines with poor neurodevelopmental outcomes at 2 years of corrected age. Short-term exposures to either or both drug classes did not significantly affect neurodevelopmental scores. However, follow-up beyond 2 years was not reported, and a subgroup analysis of mechanically ventilated patients was not conducted (33).

Compared to midazolam monotherapy, the combination of midazolam and fentanyl can provide effective analgosedation without adversely affecting vital signs and hemodynamics in neonates receiving mechanical ventilation.

## 5 Limitations

Neonatal fentanyl and midazolam exposures may affect neurodevelopmental outcomes. Consequently, we note that the lack of long-term neurodevelopmental follow-up is a limitation of this study. With the establishment of our department's outpatient follow-up clinic, long-term follow-up and development tracking will be conducted for such children in the future.

## 6 Conclusion

The combination of midazolam with fentanyl can more effectively reduce pain and ventilator parameters among mechanically ventilated neonates than midazolam monotherapy, without an increased incidence of adverse reactions or worsened discharge outcomes.

## Data Availability

The raw data supporting the conclusions of this article will be made available by the authors, without undue reservation.

## Appendix table

**Table:**

Drug Information		
Drug	Manufacturer	Drug Batch Number
Midazolam	Jiangsu Nhwa Pharmaceutical Co., Ltd	SFDA H10980025
Fentanyl	Yichang Renfu Pharmaceutical Co., LTD	SFDA H42022076
